# The Regulatory Network and Role of the circRNA-miRNA-mRNA ceRNA Network in the Progression and the Immune Response of Wilms Tumor Based on RNA-Seq

**DOI:** 10.3389/fgene.2022.849941

**Published:** 2022-04-26

**Authors:** Xiao-Mao Tian, Bin Xiang, Zhao-Xia Zhang, Yan-Ping Li, Qin-Lin Shi, Mu-jie Li, Qi Li, Yi-Hang Yu, Peng Lu, Feng Liu, Xing Liu, Tao Lin, Da-Wei He, Guang-Hui Wei

**Affiliations:** ^1^ Department of Urology, Children’s Hospital of Chongqing Medical University, Chongqing, China; ^2^ Ministry of Education Key Laboratory of Child Development and Disorders, Chongqing Key Laboratory of Pediatrics, National Clinical Research Center for Child Health and Disorders, China International Science and Technology Cooperation Base of Child Development and Critical Disorders, Children’s Hospital of Chongqing Medical University, Chongqing, China; ^3^ Chongqing Key Laboratory of Children Urogenital Development and Tissue Engineering, Chongqing, China

**Keywords:** wilms tumor, circular RNA, ceRNA, RNA sequencing, prognosis, biomarker, immune microenvironment

## Abstract

Circular RNA (circRNA), which is a newly discovered non-coding RNA, has been documented to play important roles in miRNA sponges, and the dysregulation of which is involved in cancer development. However, circRNA expression profiles and their role in initiation and progression of Wilms tumor (WT) remain largely unclear at present. Here, we used paired WT samples and high-throughput RNA sequencing to identify differentially expressed circRNAs (DE-circRs) and mRNAs (DE-mRs). A total of 314 DE-circRs and 1612 DE-mRs were identified. The expression of a subset of differentially expressed genes was validated by qRT–PCR. A complete circRNA-miRNA-mRNA network was then constructed based on the common miRNA targets of DE-circRs and DE-mRs identified by miRanda prediction tool. The Gene set enrichment analysis (GSEA) indicated that several signaling pathways involving targeted DE-mRs within the ceRNA network were associated with cell cycle and immune response, which implies their participation in WT development to some extent. Subsequently, these targeted DE-mRs were subjected to implement PPI analysis and to identify 10 hub genes. Four hub genes were closely related to the survival of WT patients. We then filtered prognosis-related hub genes by Cox regression and least absolute shrinkage and selection operator (LASSO) regression analysis to construct a prognosis-related risk score system based on a three-gene signature, which showed good discrimination and predictive ability for WT patient survival. Additionally, we analyzed the mutational landscape of these genes and the associations between their expression levels and those of immune checkpoint molecules and further demonstrated their potential impact on the efficacy of immunotherapy. qRT–PCR and western blotting (WB) analysis were used to validate key differentially expressed molecules at the RNA and protein levels, respectively. Besides these, we selected a key circRNA, circEYA1, for function validation. Overall, the current study presents the full-scale expression profiles of circRNAs and the circRNA-related ceRNA network in WT for the first time, deepening our understanding of the roles and downstream regulatory mechanisms of circRNAs in WT development and progression. We further constructed a useful immune-related prognostic signature, which could improve clinical outcome prediction and guide individualized treatment.

## Introduction

Nephroblastoma, also known as Wilms tumor (WT) is the second most common intraabdominal cancer, and the fifth most common malignancy of children, with an incidence rate of 7.1 per million children under 15 years of age (Davidoff 2009). Multiple WT treatments, including surgery, radiotherapy, and chemotherapy, have improved in recent years, and the overall survival of WT patients exceeds 80% (Dome et al., 2015). These tumors are classified into anaplastic or nonanaplastic pathological types according to the Children’s Oncology Group (COG) protocol; the former is significantly associated with aggressive pathological features and a worse prognosis (Beckwith and Palmer 1978). Unfortunately, there are still no effective molecular markers for early screening, diagnosis, and treatment. Patients with progressive or recurrent tumors have limited treatment options, leaving these patients with a poor prognosis. For patients who relapse, the 5-years survival rate has been reported to be 30–40% (Spreafico et al., 2009; Malogolowkin et al., 2013). Furthermore, treatment-related complications remain a challenging issue for WT patients (Wong et al., 2016; Neu et al., 2017; Reulen et al., 2017). Balancing therapeutic benefits and side effects is a dual challenge for WT patients (Green 2013). Accumulating studies have shown that the prognosis depends not only on the stage and pathological type but also on specific molecules (Treger et al., 2019). Hence, it is important to understand the molecular mechanisms involved in WT progression and to identify effective biomarkers to improve treatment efficacy and overall prognosis.

Noncoding RNAs (ncRNAs), which make up more than 98% of the human genome, play an essential role in gene expression and regulation (Slack and Chinnaiyan 2019). Circular RNAs (circRNAs), a newly discovered class of ncRNAs, are localized primarily in the cytoplasm, suggesting that they play major roles in the posttranscriptional regulation of gene expression (Salzman et al., 2012). Studies on circRNAs have revealed that they are widely expressed in various organisms, characterized by high expression, stability, and conservatism. Thus, circRNAs have become a research hotspot in a wide variety of human diseases and cancers. Numerous studies have revealed that circRNAs play critical roles in many diseases, especially in the field of cancer research ( Memczak et al., 2013; Zhong et al., 2018 ). CircRNAs are highly enriched in binding sites for microRNAs (miRNAs) and thus can act as sponges to regulate miRNA function by absorbing miRNAs (Zhong et al., 2018). Emerging studies have proposed a competitive endogenous RNA (ceRNA) mechanism by which circRNAs sponge miRNAs to modulate gene expression at a posttranscriptional level (Salmena et al., 2011). Notably, these ceRNA regulatory networks are significant mechanisms by which circRNAs may exert huge influences on cancer (Hansen et al., 2013; Memczak et al., 2013). A recent study found that upregulation of circCDYL could reduce WT cell proliferation, migration, and invasion. Mechanistically, circCDYL, functioning as a miRNA sponge, facilitated the circCDYL/miR-145-5p/TJP1 axis (Zhou et al., 2021). Likewise, circ0093740 promotes the growth and migration ability of WT cells by sponging miR-136/145 and upregulating DNMT3A (Cao et al., 2021). Such studies have revealed that circRNA-mediated ceRNA network participates in the regulation of development and progression in WT. However, the specific characteristics and roles of circRNAs and circRNA-associated ceRNAs in WT have not yet been reported.

**FIGURE 1:**
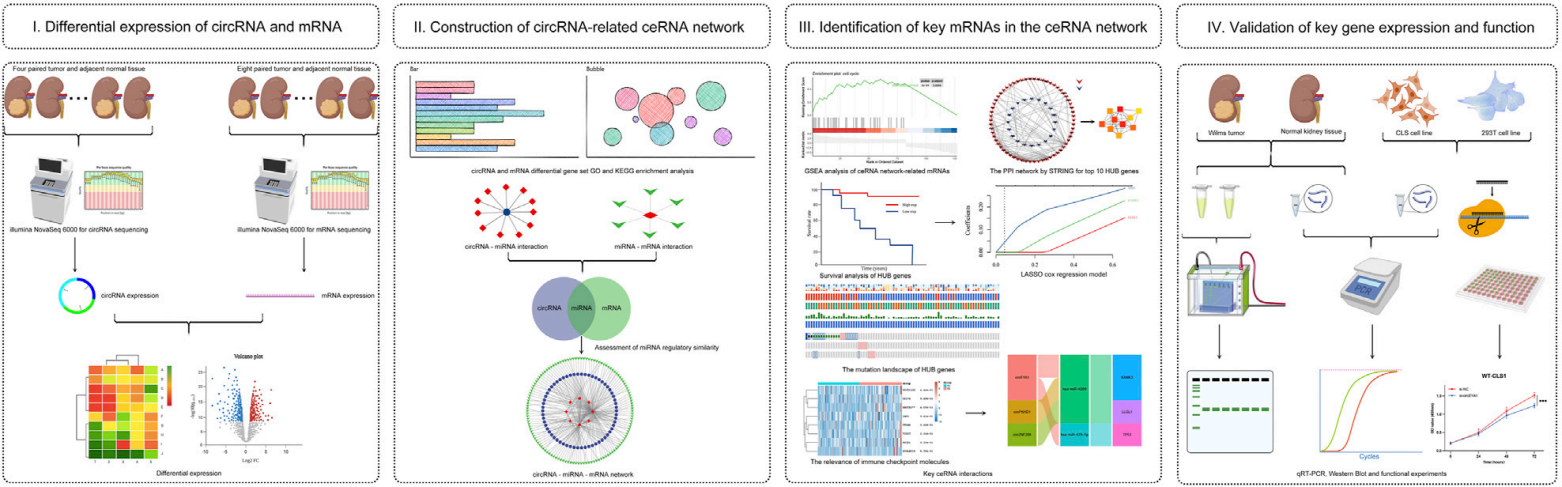
Analysis flowchart. **(I)** Identification of differentially expressed circRNA (DE-circR) and mRNA (DE-mR) by tumor tissue sequencing in Wilms tumor. **(II)** GO and KEGG pathway enrichment analyses of DE-circRs’ parental genes and DE-mRs; Construction of circRNA-miRNA-mRNA regulatory network based on same miRNA binding sites. **(III)** Identification of key mRNAs in the circRNA-related network, including GSEA, PPI network analysis, survival analysis, mutation analysis, and immune response analysis. **(IV)** Validation for key circRNAs and mRNAs in clinical samples and cell lines by qRT-PCR and western blot analysis, and functional validation of candidate circRNAs.

Recent advances in sequencing technologies have yielded a deeper understanding of circRNA and facilitated in-depth research on its formation process and mechanism. Therefore, to explore the underlying mechanism of circRNA-associated ceRNAs responsible for WT development and progression, we identified differentially expressed circRNAs (DE-circRs) and mRNAs (DE-mRs) by conducting RNA-seq of clinical tumor samples. We then constructed a dysregulated circRNA-associated ceRNA regulatory network (circRNA-miRNA-mRNA) in WT based on the sequencing data. Gene set enrichment analysis (GSEA) was performed to elucidate the potential function of the DE-mRs in the ceRNA network. To search for the key targets of the ceRNA regulatory network, a protein–protein interaction (PPI) network was constructed, and hub genes were revealed. Subsequently, we identified hub genes closely associated with disease-free survival (DFS) and constructed an mRNA-based prognostic signature based on Cox and least absolute shrinkage and selection operator (LASSO) regression analysis that contributed to the construction of a key ceRNA regulator subnetwork. Furthermore, we also investigated the mutational signature of key genes and the impact of the expression of these genes on the response to immunotherapy, which may provide new insight for the development of novel therapeutics for WT in the future. Employing qRT–PCR and western blotting (WB), we validated these results, which showed similar trends with the sequencing data. Besides these, we selected a key circRNA, circEYA1, for function validation. An overview of the entire procedure is shown in Figure 1. Taken together, our results highlight a circRNA-associated complex network in WT, broaden our understanding of WT pathogenesis, and provide a promising target for prognosis prediction and treatment.

## Materials and Methods

### Wilms Tumor Tissue Samples and Cell Lines

Tissues were obtained from the Children’s Hospital of Chongqing Medical University. All patients were diagnosed with WT according to the COG protocol (Ko and Ritchey 2009) (patients received no chemotherapy before surgery). Patients were excluded if other pathological findings were reported. For the present study, eight randomly selected pairs of normal and corresponding tumor tissues were subjected to mRNA expression analysis. To acquire complete circRNA expression profile data, circRNA sequencing was performed on four other pairs of tissues. The other twenty pairs of tissues were frozen and stored in liquid nitrogen until validation was performed. Local research ethics committee approval was granted for the study, and all patients or guardians gave informed consent.

Human WT cells (WT-CLS1) and renal epithelial cells (293T) were purchased from the American Type Culture Collection (ATCC, Manassas, United States). All cells were cultured in Dulbecco’s modified Eagle’s medium (DMEM, Gibco, United States) supplemented with 10% fetal bovine serum, 100 U/mL penicillin sodium and 100 μg/ml streptomycin at 37°C in a humidified incubator containing 5%.

### circRNA Sequencing

The total RNA samples of four paired WT samples were treated with the RiboZero rRNA Removal Kit (Epicenter, WI, United States) to remove rRNA, according to the manufacturer’s instructions. Next, the rRNA-depleted and RNase R-digested RNA samples were fragmented, and cDNA was synthesized with random primers. The PCR amplification products of cDNA were purified, and then the libraries were quality controlled and sequenced with a NovaSeq6000 instrument (Illumina, San Diego, CA, United States). The sequencing results were performed using double-ended sequencing, so each sample contained two fastq files. The sequencing quality of each fastq file was assessed using FastQC software (http://www.bioinformatics.babraham.ac.uk/projects/fastqc/). To acquire high-quality clean reads for analysis, sequences were quality-filtered with fastp (Chen et al., 2018). Then, the circRNAs were detected and identified by CIRI software (Gao et al., 2015).

Reference genomes (GRCH38/hg38) were obtained from the UCSC genome browser (http://genome.ucsc.edu/). In the first step, FASTQ reads that aligned contiguously and full length to the genomes by TopHat2 were discarded. Next, from the unmapped reads, we extracted 20-nt regions from both ends and aligned them independently to find unique anchor positions within spliced exons by TopHat2 again. The alignment of anchors in the reversed orientation (head-to-tail) indicated circRNA splicing. Using the ANNOVAR database, we mapped the splicing ends of each circRNA to the genomic regions and then compared the results to the RefSeq and UCSC databases to annotate the functional elements of circRNAs. The above results and the position relationship of circRNAs on the chromosome were recorded, and the data were matched with data from the circBase database (Glažar et al., 2014), which revealed which circRNAs were known and which circRNAs were newly predicted. Circos software was used to construct the circos figure of circRNA global presentation (Krzywinski et al., 2009).

### mRNA Sequencing

Eight paired WT samples were randomly selected for paired-ended mRNA sequencing. Library construction and sequencing were performed at Shanghai Sinomics Corporation. Total RNA was isolated using the RNeasy Mini kit (Qiagen, Germany). Paired-end libraries were synthesized by using the TruSeq™ RNA Sample Preparation Kit (Illumina, United States) following the TruSeq™ RNA Sample Preparation Guide. Briefly, poly-A-containing mRNA molecules were purified using poly-T oligo-attached magnetic beads. Following purification, the mRNA was fragmented into small pieces using divalent cations at 94°C for 8 min. The cleaved RNA fragments were copied into first-strand cDNA using reverse transcriptase and random primers. This was followed by second-strand cDNA synthesis using DNA polymerase I and RNase H. These cDNA fragments were then subjected to an end repair process, the addition of a single “A” base, and ligation of the adaptors. The products were then purified and enriched via PCR to create the final cDNA library. Purified libraries were quantified with a Qubit^®^ 2.0 Fluorometer (Life Technologies, United States) and validated with an Agilent 2100 bioanalyzer (Agilent Technologies, United States) to confirm the insert size and calculate the molar concentration. Clusters were generated by cBot (with the library diluted to 10 p.m.) and then sequenced on the Illumina NovaSeq 6000 (Illumina, United States). Additionally, the quality of sequencing data was assessed using FastQC software, and raw reads were quality filtered using FASTX-Toolkit (http://hannonlab.cshl.edu/fastx_toolkit/). Finally, clean reads were mapped to the reference genome (GRCh38.91) using HISAT2 software (Kim et al., 2015). RNA- seq data were uploaded into the gene expression omnibus database (https://www.ncbi.nlm.nih.gov/geo/) (Accessionnos. GSE197046 and GSE197047).

### Quantification and Differentially Expressed circRNAs and mRNAs

For mRNA expression analysis, we used fragments per kilobase per million mapped reads (FPKM) to evaluate the expression levels of individual genes, which was calculated as follows: $FPKM=\frac{total\;exon\;fragments}{mapped\;reads\;\left(millions\right)\times exon\;length\;\left(kb\right)}$ . Stringtie software was used to count the fragment within each gene (Buchfink et al., 2015), and the trimmed mean of M values (TMM) algorithm was used for normalization. Then, FPKM values were calculated, and differential gene expression analyses were computed with the edgeR package (Robinson et al., 2010). The obtained *p* values of group comparisons were adjusted for multiple testing using the Benjamini–Hochberg correction, and the fold-change was calculated according to the FPKM value. Finally, both |logFC| > 2 and adjusted *p* value <0.05 were used as screening standards to identify differentially expressed mRNAs.

For circRNA expression analysis, we used the number of back-spliced reads for circRNA quantification. Fold change (FC) was calculated according to spliced reads per billion mappings (SRPBM) (Li et al., 2015), which was defined as follows: $SRPBM=\frac{number\;of\;circular\;reads}{total\;mapped\;reads\;\left(units\;in\;billion\right)}$ . Additionally, we adopted the edgeR package to analyze the differences in the expression of circRNAs between WT tissues and normal kidney tissues. DE-circRs were filtered by |FC| > 2 and *p* < 0.05.

### Gene Ontology (GO) and Kyoto Encyclopedia of Genes and Genomes (KEGG)

According to their position relationship on the chromosome, information on the protein-coding gene corresponding to the circRNA can be obtained, which is defined as the circRNA host gene. To investigate the potential biological processes of the circRNAs in the ceRNA network, GO and KEGG pathway enrichment analyses were performed using the R package clusterProfiler (Yu et al., 2012).

### Construction of the circRNA–miRNA–mRNA Network

The ceRNA hypothesis reveals a novel regulatory mechanism between ncRNAs and coding RNAs. As stated earlier, circRNAs could inhibit target gene regulation mediated by miRNAs to indirectly regulate gene expression, serving as miRNA sponges to bind miRNAs competitively within their binding sites. In the previous steps, we obtained differentially expressed circRNAs and mRNAs. miRanda is an algorithm for the detection of potential microRNA target sites in genomic sequences (Enright et al., 2003) and was used to predict miRNA targets for the identified circRNAs and mRNAs (the screening conditions were a total score of complementary sequence >140 and a total energy of thermodynamic stability < −20 kcal/mol). In these interaction networks, the circRNAs and mRNAs were linked by shared miRNA targets. Finally, the core-regulatory ceRNA network was constructed according to the same expression trends of circRNAs and mRNAs, and the network was visualized by Cytoscape software (v.3.8.2) (Shannon et al., 2003).

### Gene Set Enrichment Analysis

GSEA is a computational method that determines whether an a priori defined set of genes shows statistically significant, concordant differences between two biological states (e.g., tumor and normal tissue) (Subramanian et al., 2005). Potential pathways and biological processes involved in the DE-mRs from the ceRNA network were analyzed using GSEA on the basis of Molecular Signatures Database v7.4. We used C5 curated gene sets to analyze the enriched functional GO terms and KEGG pathways. Immunologic signature gene sets were used to identify particular biological processes or molecular functions associated with cell states and perturbations within the immune system. The enrichment analysis was conducted using the default weighted enrichment statistics method to acquire the normalized enrichment score (NES). |NES| > 2 and *p* < 0.05 were considered to indicate statistical significance.

### Construction of the Protein-Protein Interaction (PPI) Network and Hub Gene Analysis

The PPI network among DE-mRs in the ceRNA network was constructed using the Search Tool for the Retrieval of Interacting Genes/Proteins (STRING) online database (Szklarczyk et al., 2016; Szklarczyk et al., 2019). Subsequently, Cytoscape software was used to visualize the PPI network, and the cytoHubba algorithm (Chin et al., 2014) was used to identify the top ten hub genes from the PPI network.

### Establishment of a Prognostic Hub Gene Signature Based on the ceRNA Subnetwork

The raw counts of RNA-seq data (level 3) and corresponding clinical information of patients providing 136 WT samples were obtained from the Therapeutically Applicable Research To Generate Effective Treatments (TARGET) database (https://ocg.cancer.gov/programs/target), which is the largest database of childhood cancer data. For survival analysis, patients were grouped into high or low expression groups according to the median expression of the hub DE-mRs. Disease-free survival was the primary endpoint, and we estimated the prognostic value of hub DE-mRs using the Kaplan-Meier method and Cox regression analysis based on expression profiles from the TARGET dataset. The LASSO regression algorithm for feature selection was applied to screen the optimal gene combination for constructing the risk signature. The dotted line represents the median risk score and divides the patients into low-risk and high-risk groups. Afterward, Kaplan–Meier survival analysis and time ROC analysis were performed to verify the predictive accuracy of this gene signature. Ultimately, a circRNA-miRNA-mRNA ceRNA network with hub prognostic DE-mRs as the core was reconstructed based on the prognostic hub gene signature. The above analysis used the R software (version 4.0.3) packages ‘survival’, “survminer,” and “glmnet”.

### Gene Mutation and Immune Response Analysis of Gene Signature

To maximize the use of the established regulatory network, we further analyzed the gene mutation landscape of the gene signature and their effect on the potential response to immune checkpoint blockade (ICB) therapy. Data on copy number alterations and mutations were downloaded from cBioPortal (http://www.cbioportal.org/). According to the Kaplan–Meier curves, we also evaluated the effects of the mutated hub genes on the prognosis of WT patients. To investigate the effects of the key hub genes on the immune response, raw counts of RNA-seq data (level 3) from WT were obtained from the TARGET dataset. IGLEC15, TIGIT, CD274, HAVCR2, PDCD1, CTLA4, LAG3, and PDCD1LG2 were selected as immune checkpoint-relevant transcripts, and the expression values of these eight genes were extracted. According to the expression level of hub genes, the samples were divided into a high expression group and a low expression group, and the immune checkpoint gene expression level was compared between different groups. Immunotherapy was first proposed in the late 19th century; it refers to a new treatment method harnessing the immune system’s potential to eliminate cancer cells and has revolutionized the human cancer therapy field. The response of tumors to immune checkpoint inhibitors was predicted according to the Tumor Immune Dysfunction and Exclusion (TIDE) score, calculated based on a computational algorithm based on the gene expression profile (Jiang et al., 2018). The TIDE algorithm can use gene expression information to predict the sensitivity of cancer to immune checkpoint therapy, which integrates data on two tumor immune escape mechanisms (the induction of T cell dysfunction in tumors with high infiltration of cytotoxic T lymphocytes and the prevention of T cell infiltration in tumors with low cytotoxic T lymphocyte levels). Briefly, the higher the score, the worse the treatment efficacy and prognosis.

### Validation of RNA-Seq Results by qPCR and Key Gene Expression by WB

For real-time PCR, RNA from each tissue was isolated using TRIzol reagent (Invitrogen, Carlsbad, CA, United States). Total cellular RNA was extracted using the Simply P Total RNA Extraction Kit (BioFlux, China) according to the manufacturer’s instructions. RNA concentrations were determined using a NanoDrop One instrument (Thermo Fisher Scientific, Waltham, MA, United States). Reverse transcription of circRNA and mRNA was carried out using the PrimeScript™ RT reagent Kit (TaKaRa, Japan), and cDNA was amplified and quantified using the TB Green^®^ Premix Ex Taq™ II kit (TaKaRa, Japan). Real-time PCR was performed on a CFX Connect Real-Time PCR Detection System. For each sample, at least three biological repeats and three technical replicates were used. GAPDH was used for the normalization of mRNA and circRNA. The expression of target genes was based on the formula 2^−∆∆Ct^. The primers used are listed in Supplementary File S1.

Briefly, for WB, proteins were extracted from tissue with RIPA lysis buffer (Thermo Scientific). The protein concentration was quantified by a BCA assay. After quantification, total proteins were separated by SDS–PAGE and then transferred to PVDF membranes (Millipore, United States). Next, the samples were blocked for 10 minutes using QuikBlockTM Western blocking buffer (Beyotime). The membranes were washed three times for 10 min each in TBST before and after blocking and were then incubated overnight at 4°C with antibodies against p53 (1:1,000, ZEN-BIOSCIENCE) and GAPDH (1:1,000, ZEN-BIOSCIENCE). Immediately after, the membranes were incubated with horseradish peroxidase-conjugated goat anti-rabbit IgG or goat anti-mouse IgG (1:10,000, Thermo Scientific). Finally, the protein signals were detected using a Super ECL Kit (SORFA). Images were obtained with a ChemiDoc™ Touch Imaging System (BIO-RAD) and analyzed with Image Lab software (BIO-RAD).

### Functional Validation of the Selected circRNA

CircEYA1 (chr8:71244603-71271897) was selected for further functional validation. In briefly, the siRNA targeting the back-splice site of circEYA1 and the negative control siRNA were designed and synthesized by Hanbio (Shanghai, China). Lipofectamine 2000 (Invitrogen, United States) was used during transfection. According to the manufacturer’s instructions, CCK8 assay (MCE, HY-K0301) was used to detect the viability of cell, and the scratch wound healing assay was employed to investigate the cell migration. Cell invasion was detected by Transwell assay in a 24-well plate Transwell chamber (Falcon, 353097, United States) with matrix gel (Biozellen, B-P-00002-4, China) in advance. Cell cycle phase distribution was determined by flow cytometry using BD cell cycle detection kit.

### Statistical Analysis

Categorical variables were expressed as percentages and analyzed using the chi-square test. A *t* test and the Wilcox test were used to analyze the differences between two groups. A paired-samples *t* test was used to analyze differences in gene expression between WT tissues and paired adjacent tissues. The experiments were performed thrice independently. GraphPad Prism version 8 software (GraphPad Prism Software Inc., La Jolla, CA) was used to analyze the experimental results. All bioinformatics analyses and R packages were performed using R software version 4.0.3 (The R Foundation for Statistical Computing, 2020). Single, double and triple asterisks and ns indicate the significance level (*, *p* < 0.05; **, *p* < 0.01; ***, *p* < 0.001; and ns, no significance, respectively).

## Results

### Identification and Differentially Expressed circRNAs and mRNAs in Wilms Tumor

**FIGURE 2:**
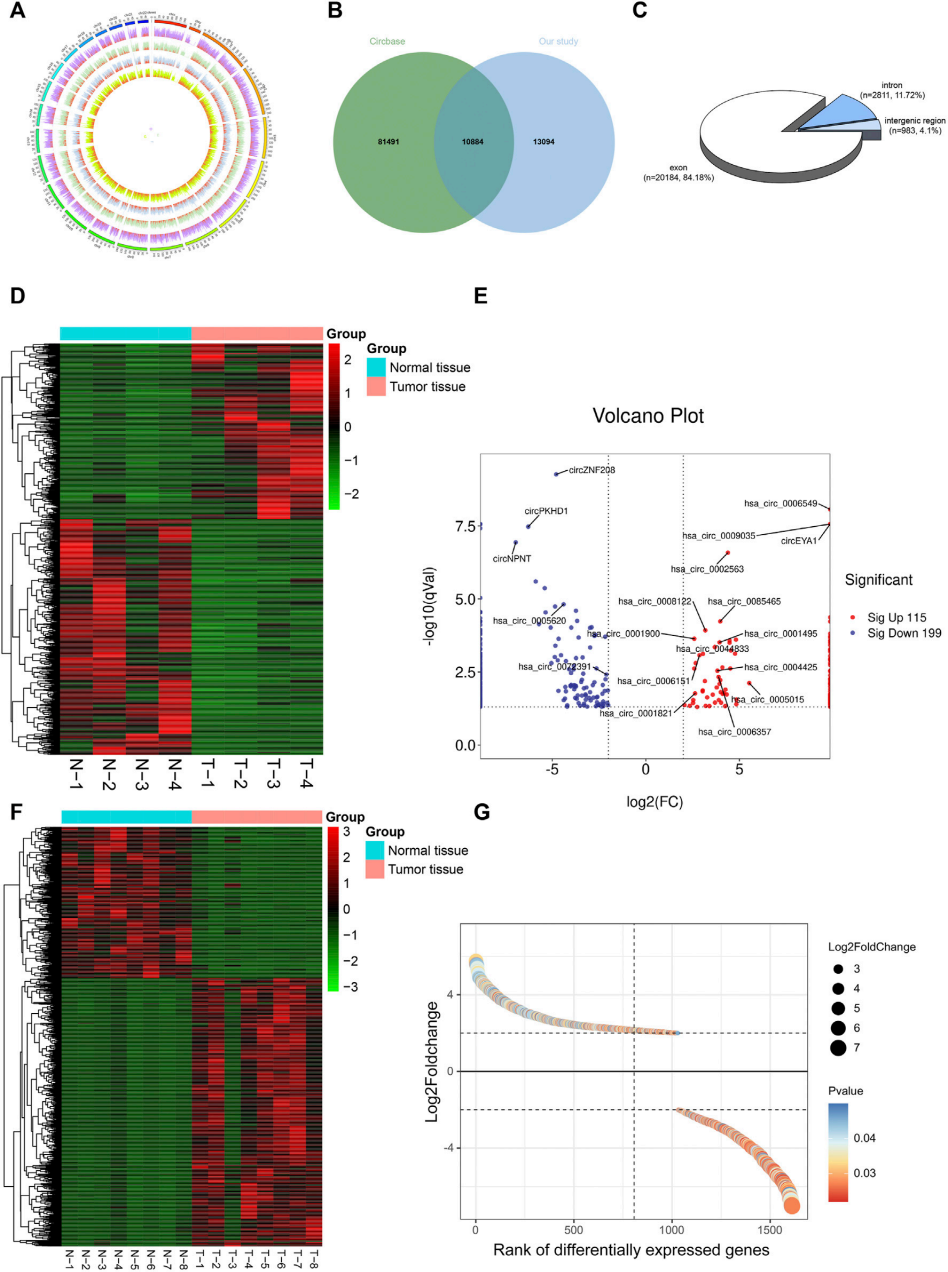
Identification of DE-circRs and DE-mRs by tumor tissue sequencing in Wilms tumor. **(A)** The circos figure of circRNA global presentation, the outer circle represents chromosomes, inner circle represents samples, and red fraction of each sample represents circRNA junction reads. **(B)** Wayne figure of circRNA sequencing data versus circBase database. **(C)** circRNAs classification. **(D)** Heatmap and clustering analysis of DE-circRs. Each row represents one circRNA and each column represents one sample; -2, -1, 0, 1, and 2 represent fold change. Red indicates high expression and green represents low expression. T represent tumor samples, N represent normal samples. **(E)** Volcano plot of DE-circRs. Red dots represent up-regulated circRNAs and blue dots represent down-regulated circRNAs. The horizontal dotted lines represent an adjusted *p*-value of 0.05, and the vertical dotted line represent a logFC of 2-fold. Selected candidates for subsequent qRT-PCR validation are annotated on figure. **(F)** Heatmap and clustering analysis of DE-mRs. **(G)** Gene-rank figure of DE-mRs. The DE-mRs were sorted by their fold change. The horizontal dotted lines represent a logFC of 2-fold. DE-circRs: differentially expressed circRNAs, DE-mRs: differentially expressed DE-mRs.

**FIGURE 3:**
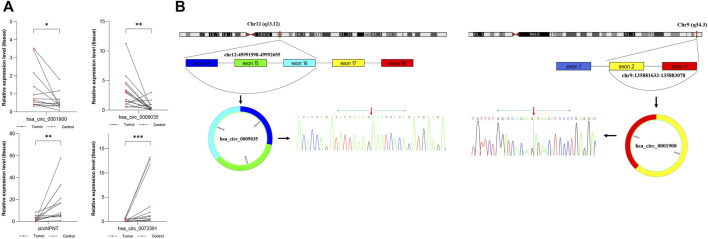
Verification of dysregulated circRNAs. **(A)** Expression level of hsa_circ_0001900, hsa_circ_0009035, circNPNT, and hsa_circ_0072391. Results were analyzed by paired *t*-test.^*^: *p* < 0.05, ^**^: *p* < 0.01, ^***^: *p* < 0.001. **(B)** The genomic loci of hsa_circ_0001900 or hsa_circ_0009035 and schematic representation of their formation. The expression of hsa_circ_0001900 or hsa_circ_0009035 was validated by qRT-PCR using a spanning junction primer followed by Sanger sequencing. The red arrow represents head-to-tail splicing sites.

To construct a circRNA profile database of WT patients, we collected four paired clinical tumor tissues to perform high-throughput circRNA sequencing. The sequencing results are detailed in Supplementary File S2. Most of the clean reads used for circRNA identification were mapped to the reference genome and a total of 23,978 circRNAs were detected (Figure 2A). A total of 10,884 circRNAs were identified (Figure 2B) when compared with the known database circBase (http://www.circbase.org/). In addition, chromosome distribution analysis showed that circRNAs were widely distributed across chromosomes 1 and 10-19. Of these, based on the sequences, most (84.18%) consisted of exons, 11.72% of introns, and the remaining 4.10% mapped to intergenic regions (Figure 2C). The expression of DE-circRs was shown via a heatmap (Figure 2D), and hierarchical clustering revealed that most DE-circRs had decreased expression in WT versus normal samples (a total of 1755 DE-circRs were identified, including 749 upregulated and 1,006 downregulated circRNAs in WT). Furthermore, we defined the cutoff as |logFC| > 2 and adjusted *p* < 0.05 to narrow down the DE-circRs and excluded circRNAs derived from intergenic regions. A volcano plot was then used to show the distribution of DE-circRs (Figure 2E, Supplementary File S3). To validate our circRNA-seq data, we randomly selected 4 DE-circRs (detailed characteristics are shown in Supplementary File S4) and designed divergent primers for specific back-splicing sites. Consistent with the sequencing data, the qRT-PCR results showed similar trends in WT tissues (Figure 3A). To verify the back-splice junction sequences, 2 randomly selected DE-circR qRT–PCR-amplified products were sent for Sanger sequencing, and the sequencing results confirmed the back-splice junction sites (Figure 3B). Similarly, DE-mRs were detected in an additional eight paired WT tissues; 1,030 genes upregulated and 582 were downregulated (Figures 2F,G).

### Gene Ontology and Kyoto Encyclopedia of Genes and Genomes Pathways of the Parental Genes of Differentially Expressed-circRs and Differentially Expressed-mRs

**FIGURE 4:**
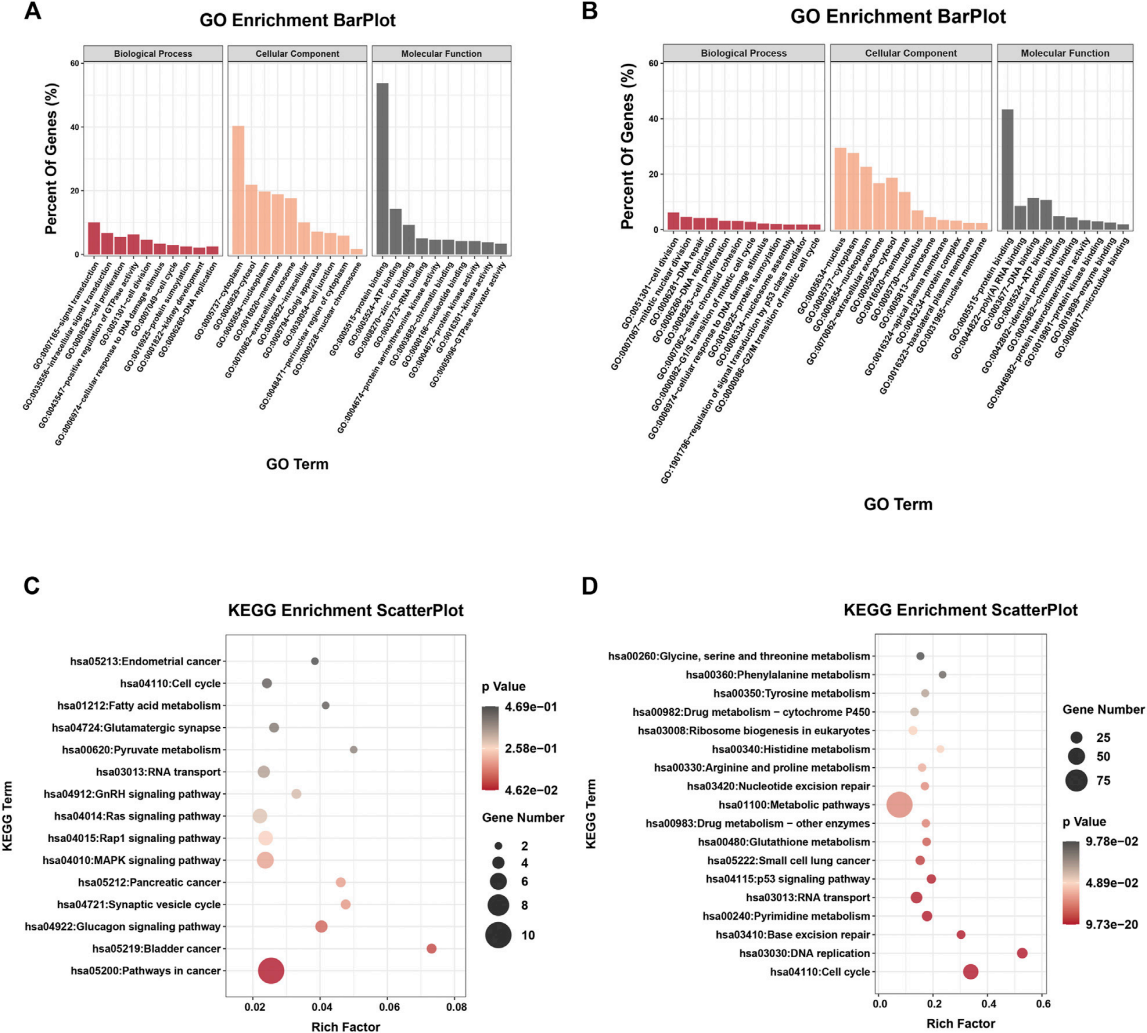
GO and KEGG enrichment analysis of the parental genes of DE-circRs and DE-mRs. **(A)** GO enrichment analysis for the parental genes of DE-circRs. **(B)** GO enrichment analysis for DE-mRs. **(C)** KEGG enrichment analysis for the parental genes of DE-circRs. **(D)** KEGG enrichment analysis for DE-mRs. GO, Gene Ontology; KEGG, KyotoEncyclopedia of Genes and Genomes.

To investigate the possible biological functions of DE-circRs and DE-mRs in WT, GO and KEGG pathway enrichment analyses of the host genes of DE-circRs and DE-mRs were performed. The results of enrichment analysis of DE-circR host genes are shown in Figures 4A,C. GO annotation analysis showed that the DE-circR host genes were mainly located in the “cytoplasm” and “extracellular exosome” and involved in “cell division”, “cell proliferation”, “cell cycle” and other processes. KEGG pathway enrichment analysis revealed that the DE-circR host genes were mainly enriched in multiple cancer-related pathways. In addition, GO and KEGG pathway function enrichment analyses were also employed for these DE-mRs. For GO functional annotation (Figure 4B), multiple biological processes correlated with malignant phenotypes were significantly enriched (for example, “DNA replication”, “cell division”, “cell cycle”, “cell proliferation”, and so forth). KEGG pathway enrichment analysis (Figure 4D) showed that the DE-mRs were mainly concentrated in cell growth, proliferation, and metabolism signaling pathways, and some cancer-related signaling pathways, such as the p53 signaling pathway and multiple metabolic pathways were also enriched. These results are consistent with those of DE-circRs.

### Construction of the circRNA-miRNA-mRNA Network

**FIGURE 5:**
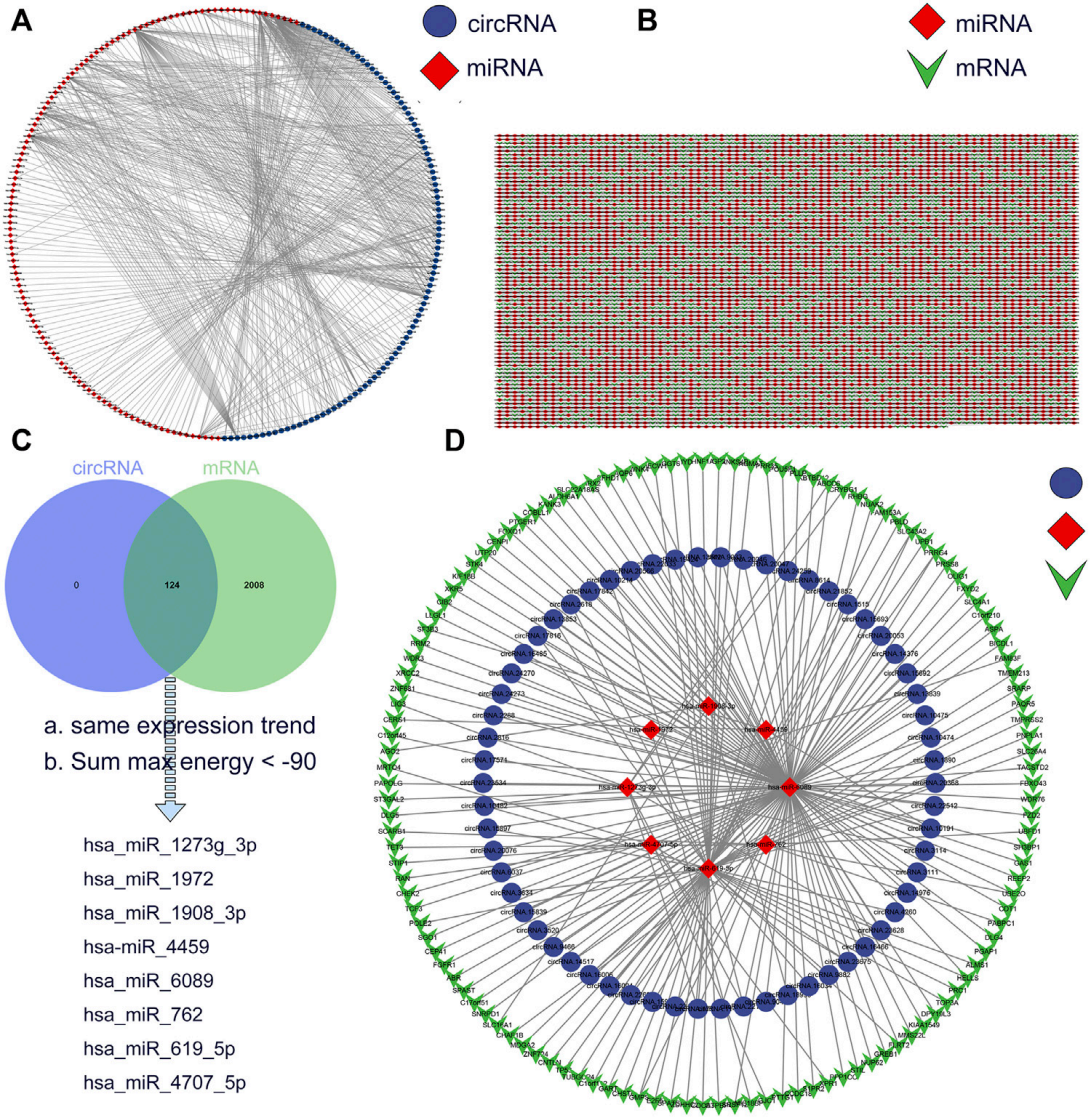
Construction of a circRNA-miRNA-mRNA ceRNA network in Wilms tumor. **(A)** circRNA-miRNA regulatory network. Blue circles represent circRNA and red rhombi represent miRNA. **(B)** miRNA-mRNA regulatory network. Red rhombi represent miRNA and green triangles represent mRNA. **(C)** The intersection of miRNA target between DE-circRs and DE-mRs. **(D)** The ceRNA regulatory network. Blue circles represent circRNA, red rhombi represent miRNA, and green triangles represent mRNA.

CircRNAs act as miRNA sponges and subsequently block the function of their corresponding miRNAs (Hansen et al., 2013). We used miRanda to predict miRNA-bound DE-circRs and the target genes of DE-miRs, and the circRNA-miRNA interactions and the miRNA-mRNA interactions were assessed by using Cytoscape, as shown in Figures 5A,B. Then, we integrated the circRNA-miRNA pairs and miRNA-mRNA pairs to construct a complete circRNA-related regulatory network based on these same miRNA binding sites. According to the theory of ceRNA, we set the screening condition as synergistic expression trends between DE-circRs and DE-mRs and sum max energy < −90 (the greater the absolute value was, the higher the correlation). Finally, a total of 62 DE-circRs, 8-DEmiRs, and 127 DE-mRs were eventually incorporated into the precise ceRNA regulatory network by applying Cytoscape software (Figures 5C,D).

### Gene Ontology, Kyoto Encyclopedia of Genes and Genomes and Gene Set Enrichment Analysis Analyses of Differentially Expressed-mRs in ceRNA Regulatory Network

**FIGURE 6:**
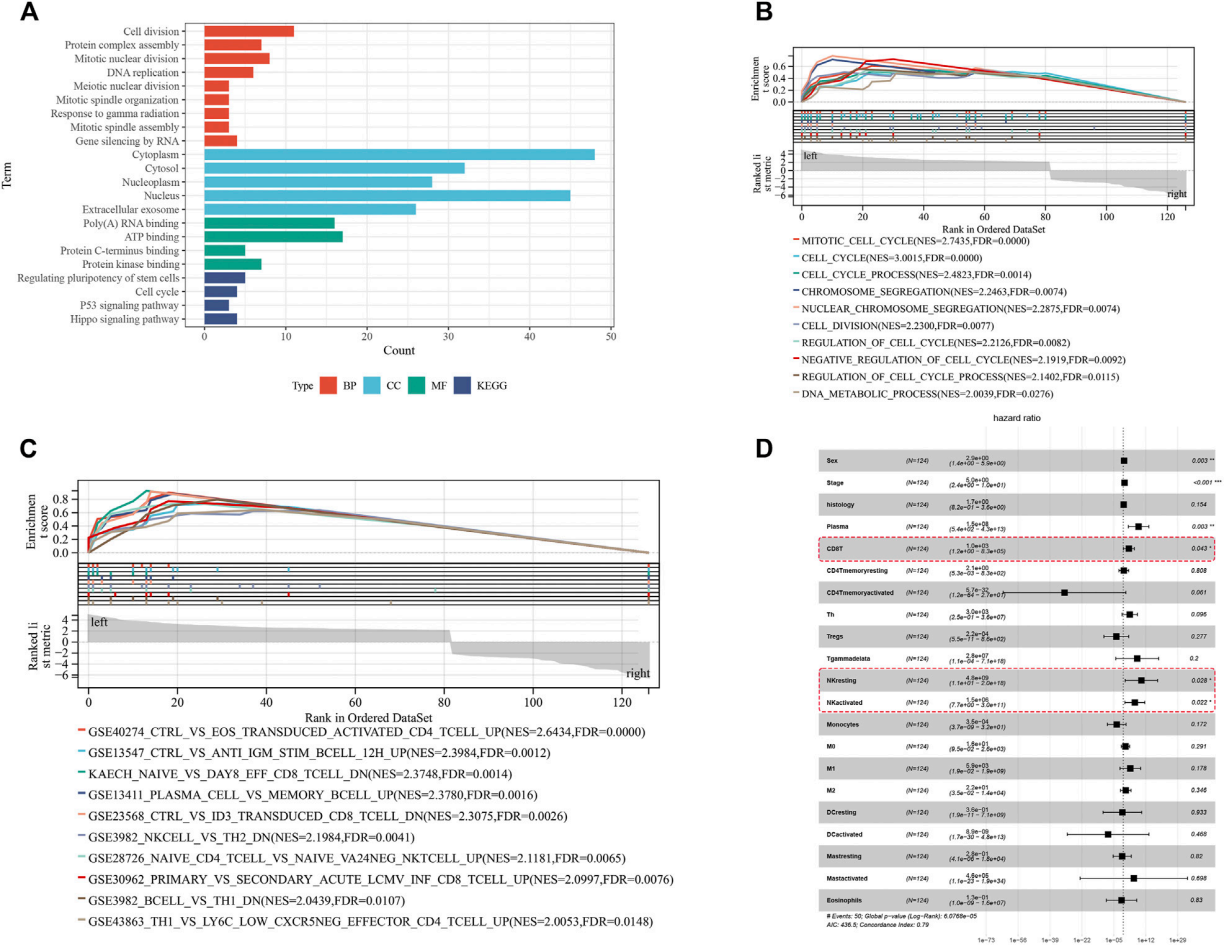
Functional enrichment analysis of DE-mRs in the ceRNA network. **(A)** GO and KEGG enrichment analysis of DE-mRs in the ceRNA network. **(B)** Enrichment plot of GSEA analysis of DE-mRs in the ceRNA network based on ‘C5’ curated gene sets. **(C)** Enrichment plot of GSEA analysis of DE-mRs in the ceRNA network based on immunologic signature gene sets. **(D)** Forest plot showing the prognostic associations of subsets of immune cells in Cox regression analysis. ^*^: *p* < 0.05, ^**^: *p* < 0.01, ^***^: *p* < 0.001.

To better understand the biological functions of DE-circRs in the ceRNA network, we analyzed the 127 DE-mRs of the ceRNA network using GO, KEGG, and GSEA analyses. The results of GO and KEGG enrichment analysis of DE-mRs are shown in Figure 6A. For biological processes (BPs), the top three enriched terms were “cell division”, “mitotic nuclear division” and “DNA replication”; for cellular components (CCs), the top enriched terms were “cytoplasm”, “cytosol” and “nucleus”; and for molecular function (MF), the top enriched terms were “poly(A) RNA binding”, “ATP binding” and “protein kinase binding”. “Cell cycle”, “p53 signaling pathways” and “Hippo signaling pathway” were significantly enriched in the KEGG pathway analysis. Because small, but coordinated, changes in gene expression can have profound biological effects, GSEA is designed to detect coordinated differences in the expression of predefined sets of functionally related genes to avoid ignoring the biological characteristics and functions of some genes. The GSEA results highlighted that these genes were mostly associated with cell cycle-related signaling pathways (Figure 6B). Notably, enrichment analysis of gene sets that represent cell states and perturbations within the immune system revealed that these DE-mRs were related to NK cell and CD8^+^ T activity (Figure 6C). Based on the TARGET database, univariate Cox regression analysis showed a significant association of NK or CD8^+^ T cell activity with WT patient prognosis (Figure 6D). These results suggested that these DE-mRs are closely related to tumorigenesis and progression and, to some extent, are involved in the cell cycle and immune response.

### Identification of Disease-Free Survival-Related Hub Differentially Expressed-mRNAs

**FIGURE 7:**
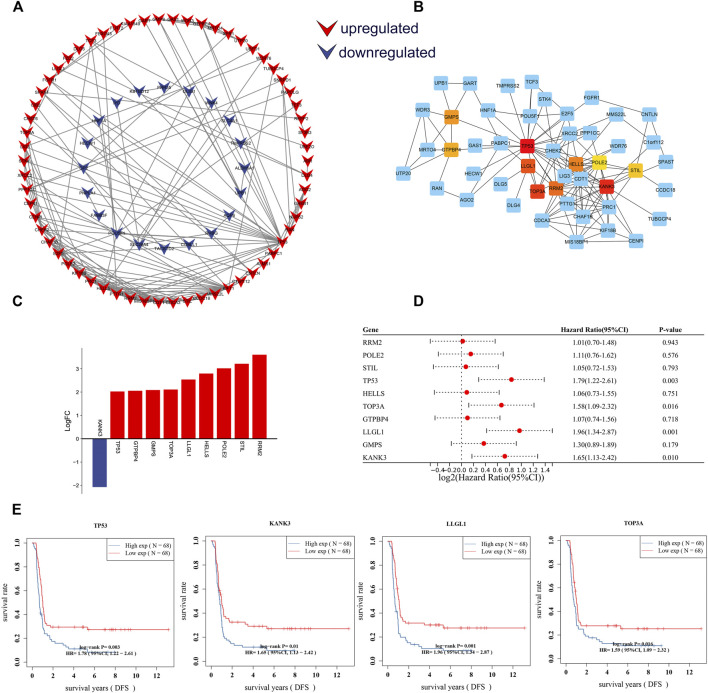
Construction of the PPI network and identification of DFS-related hub genes. **(A)** PPI network of DE-mRs in the ceRNA network. **(B)** The PPI network for top 10 hub genes identified by cytoHubba. **(C)** Column chart of expression changes of the top 10 hub genes. Red column indicate up-regulated (*X*-axis > 0) genes, blue column indicate down-regulated (*X*-axis < 0) genes. **(D)** Forest plot showing the prognostic associations of hub genes in Cox regression analysis. **(E)** Survival curves of four hub-genes that are significantly related to the prognosis of Wilms tumor patients. The horizontal axis shows the follow-up time in years, and the vertical axis shows the probability of disease-free survival. DFS: disease-free survival.

To further identify key mRNAs in the circRNA-related ceRNA network, mRNAs of the ceRNA network were submitted to the STRING database. A PPI network was established by Cytoscape software including 127 nodes and 170 edges based on the STRING database (Figure 7A). Afterward, the top 10 hub genes were identified by cytoHubba, including TP53, KANK3, STIL, RRM2, HELLS, POLE2, TOP3A, LLGL1, GMPS, and GTPBP4 (Figure 7B). According to the sequencing results, all hub genes except KANK3 were upregulated (Figure 7C). Further Cox regression analysis suggested that the expression level of four hub genes was significantly correlated with DFS (Figures 7D,E), including tumor protein p53 (TP53), KN motif and ankyrin repeat domains 3 (KANK3), scribble cell polarity complex component (LLGL1) and DNA topoisomerase III alpha (TOPA3). These results imply that these four genes probably play a critical role in the progression and prognosis of WT.

### Establishment of a Prognostic Hub Gene Signature Based on the ceRNA Subnetwork

**FIGURE 8:**
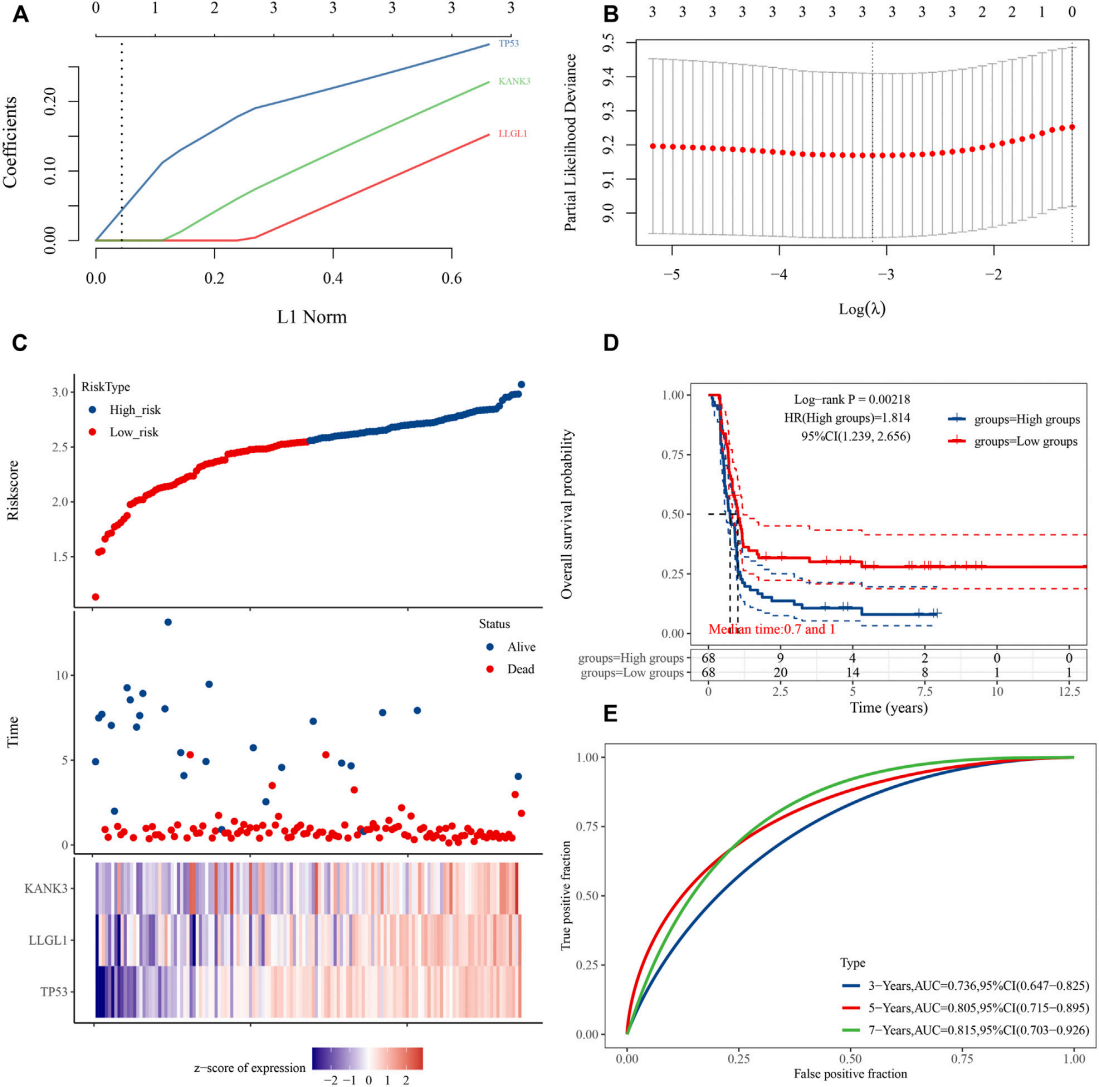
Establishment of a three-gene prognostic signature regulated by the ceRNA mechanism. **(A)** LASSO coefficient profiles of the 3 prognostic DE-mRNAs within the subnetwork. **(B)** LASSO deviance profiles of the 3 prognostic DE-mRNAs within the subnetwork. **(C)** Risk score distribution and survival status for each patient, and expression heat map of three genes corresponding to each sample above. **(D)** Comparison of prognostic differences between the high- and low-risk groups divided by signature model. **(E)** Evaluation efficacy of the signature model at 3, 5, and 7 years. Area under the curve is 0.736, 0.805, and 0.815, respectively.

**FIGURE 9:**
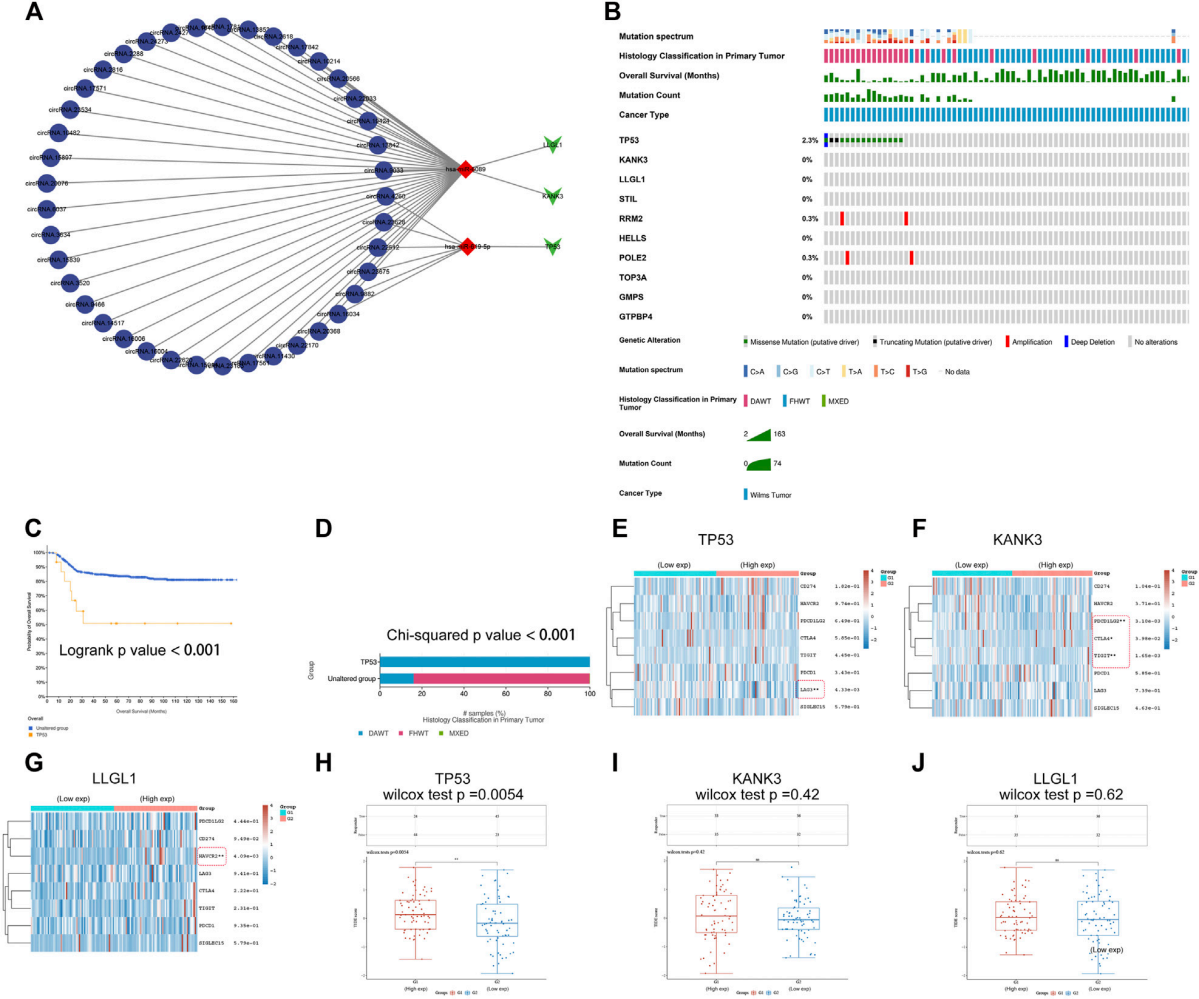
Establishment of a ceRNA sub-network and analysis of mutational landscape of gene signature and its effect on immune response. **(A)** A ceRNA sub-network based on the three-gene prognostic signature. **(B)** The mutational profiles of gene signature in Wilms tumor, data of genetic alteration were obtained from the cBioPortal for Cancer Genomics (https://www.cbioportal.org). **(C)** Comparison of survival between patients with and without TP53 mutations. **(D)** Comparison of pathological types between patients with and without TP53 mutations. DAWT: anaplasia type, FHWT: favorable histology type. **(E-G)** Comparison of gene expression of immune checkpoint-related genes between the high- and low-expression TP53, KANK3, or LLGL1 groups. Each row represents one immune checkpoint-related gene and each column represents one sample; Red indicates high expression and green represents low expression of immune checkpoint-related genes. **(H-J)** Comparison of TIDE score between the high- and low-expression TP53, KANK3, or LLGL1 groups. Tumor Immune Dysfunction and Exclusion (TIDE) score was used to predict potential tumor therapeutic response with immune checkpoint inhibitors. ^*^: *p* < 0.05, ^**^: *p* < 0.01, ^***^: *p* < 0.001.

Considering the importance of DFS in the clinical outcome of WT patients, we also constructed a prognostic gene signature for DFS. A three-mRNA signature was constructed by LASSO Cox regression analysis, and the risk prediction model of these hub genes was established and evaluated (Figures 8A,B). The results showed that high expression of TP53, KANK3, and LLGL1 in tumor tissues predicted a worse prognosis. Using the LASSO Cox regression models, we also calculated a risk score for each patient, and these patients were stratified into two groups (high risk and low risk) based on this gene set risk score (Figure 8C). Survival analysis showed that patients in the high-risk group had a poorer DFS than those in the low-risk group (Figure 8D). Next, receiver operating characteristic (ROC) analysis showed that the area under the curve (AUC) values of this model at 3-, 5- and 7-years DFS reached 0.736, 0.805, and 0.815, respectively, indicating the high accuracy of the model (Figure 8E). Finally, based on the importance of the gene signature in WT progression and prognosis, we further constructed a gene signature-related subnetwork (Figure 9A).

### Mutational Landscape of Signatures and its Effect on the Immune Response

Gene mutation is one of the hallmarks of cancer. These changes are likely to be associated with tumor progression and drug resistance and could influence the immunotherapy response. In recent years, biomarkers for diagnosis and therapy have revolutionized the treatment of cancers. To investigate mutations in the gene signature in WT, genetic alterations of the hub genes were analyzed using cBioPortal (Figure 9B). The results showed that TP53 had the highest prevalence of gene mutations. Survival analysis indicated significantly decreased DFS in the TP53-mutated group compared to the TP53-wild-type group (Figure 9C). In addition, TP53 mutation status was correlated with the anaplastic pathological subtype, which is considered a critical factor for a poor prognosis in WT patients (Figure 9D).

The application of cancer immunotherapy has gained great attention in recent years. Immune checkpoints refer to a series of molecules expressed on immune cells that can regulate the degree of immune activation and play an important role in preventing the occurrence of autoimmunity. Tumor cells escape immune surveillance and progress through different mechanisms, such as the overexpression of inhibitory immune checkpoint molecules that impair the antitumor immune response (Orabona et al., 2018). Immune checkpoint blockade (ICB) therapy has become a revolutionary immune-based treatment in cancers. The results of differential gene expression analysis showed that immune checkpoint-related genes were differentially expressed between the high and low TP53, KANK3, or LLGL1 expression groups (Figures 9E–G). Next, we further evaluated the impact of these hub genes on ICB-mediated immune responses in WT. Surprisingly, the TIDE score was significantly different between the TP53 high expression group and the TP53 low expression group (Figures 9H–J). These results suggest that the TP53 high expression subgroup that received ICB therapy exhibited worse clinical efficacy and shorter survival. The results echo the previous analysis that high TP53 expression was associated with a poorer prognosis. These data suggested that TP53 might play important regulatory roles with multiple pathways, serving as a potential target for antitumor immunotherapy in WT.

### Validation of Key circRNAs and mRNAs in Clinical Samples and Cell Lines

**FIGURE 10:**
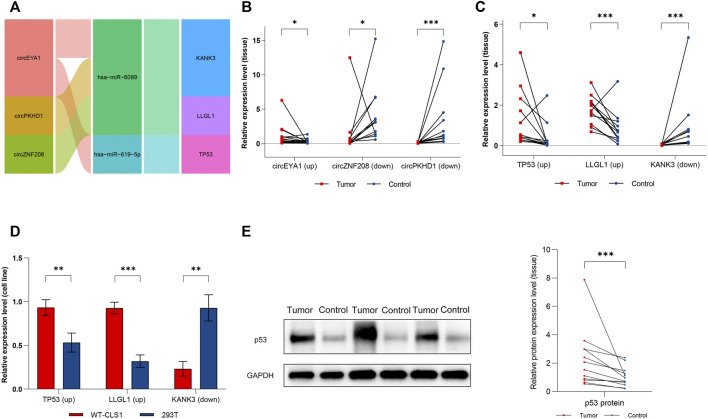
Verification of dysregulated circRNAs and mRNAs. **(A)** Sankey diagram of the ceRNA sub-network for experimental verification. **(B)** Expression level of circEYA1, circZNF208, and circPKHD1 in clinical samples. **(C)** Expression level of TP53, KANK3, and LLGL1 in clinical samples. **(D)** Expression level of TP53, KANK3, and LLGL1 in cell lines. **(E)** Expression level of P53 protein in clinical samples. Red dots represent tumor samples, blue dots represent normal samples. Results were analyzed by paired *t*-test **(B,C,E)** or *t*-test **(D)**. ^*^: *p* < 0.05, ^**^: *p* < 0.01, ^***^: *p* < 0.001.

We screened the three most distinctively expressed DE-circRs (Figure 10A, detailed characteristics are shown in Supplementary File S4) from the subnetwork for verification by qRT-PCR using clinical samples, which was consistent with our RNA sequencing results (Figure 10B). We then validated the expression of three key DE-mRs (TP53, KANK3, and LLGL1) in both clinical samples and cell lines (Figures 10C,D). Finally, we examined the expression of p53 protein in tumor samples, given its important role in WT (Figure 10E).

### Silencing of circEYA1 Suppressed Proliferation, Invasion, and Migration in WT Cells

**FIGURE 11:**
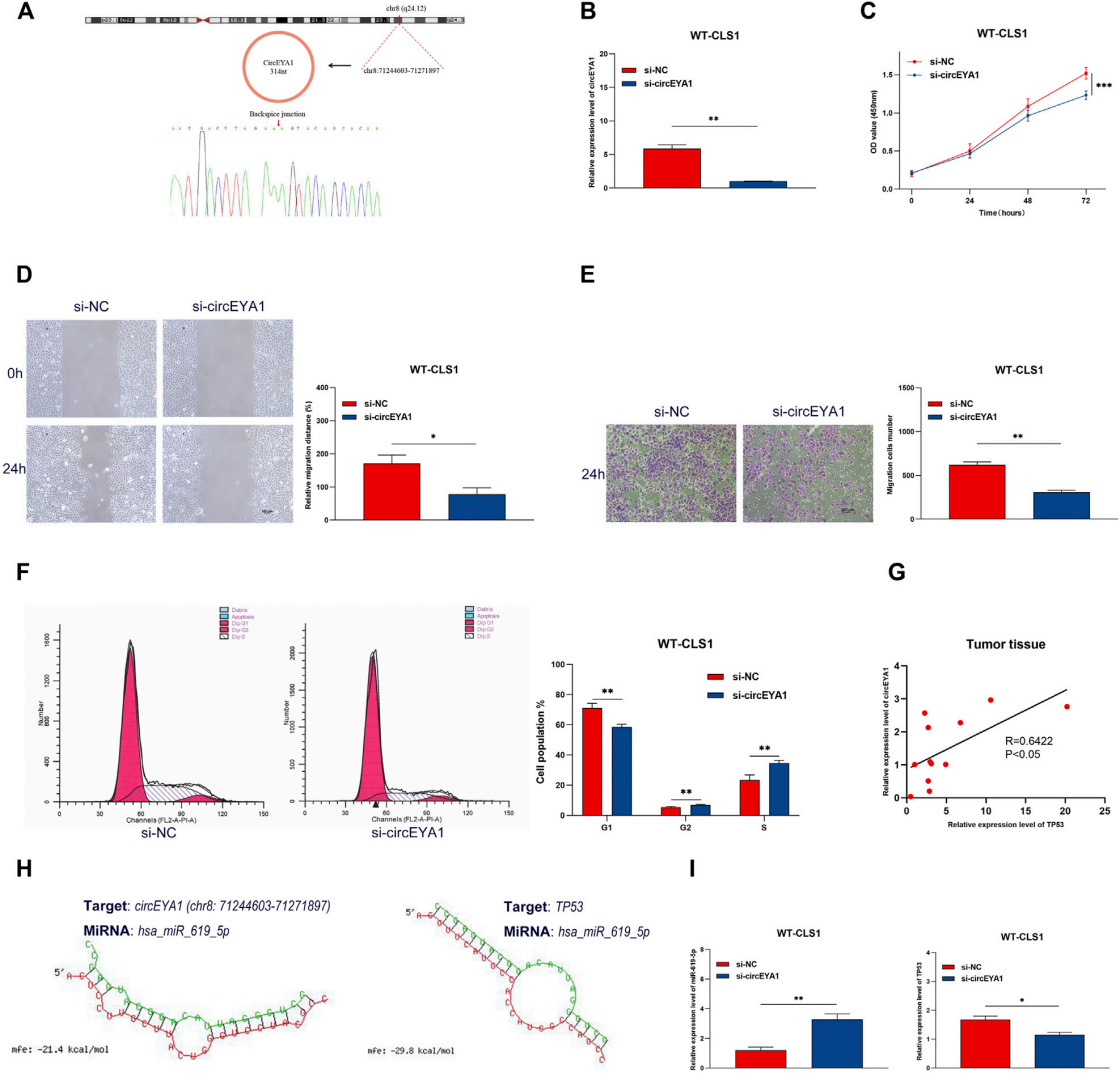
Silencing of circEYA1 suppressed proliferation, invasion, and migration in WT cells. **(A)** The backsplice junction site of circEYA1 was identified by Sanger sequencing. **(B)** qRT-PCR analysis of circEYA1 expression in WT-CLS1 cells after transfection with siRNA targeting the back-splice site. **(C)** The proliferation of WT-CLS1 cells by the CCK-8 assay. **(D–E)** The migration and invasion of WT-CLS1 cells by wound healing assays and transwell assays. **(F)** circEYA1 silencing induced cell cycle arrest at the S and G2 phases. **(G)** The relative expression of TP53 mRNA had a positive correlation with circEYA1 in WT tissue by RT-qPCR. **(H)** Possible binding sites between miR-619-5p and circEYA1 or TP53. **(I)** Changes in relative expression levels of miR-619-5p or TP53 after circEYA1 silencing in WT-CLS1 cells. ^*^: *p* < 0.05, ^**^: *p* < 0.01, ^***^: *p* < 0.001.

CircEYA1 (chr8:71244603-71271897) was selected for further functional validation. As previously described, circEYA1 is highly expressed in WT tissues compared with adjacent non-tumor tissues. CircEYA1 is a 314 nt circRNA transcript generated by back-splicing of the EYA1 gene. Sanger sequencing of PCR products demonstrated the back splice junction site (Figure 11A). To investigate the functional role of circEYA1 in WT carcinogenesis, we designed a siRNA for silencing of circEYA1. The siRNA was designed to specifically target the circEYA1 back-splice junction. Compared with the negative control siRNA, si-circEYA1 greatly decreased the circEYA1 level in WT-CLS1 cells (Figure 11B). Silencing of circEYA1 significantly suppressed the proliferation ability of WT cells through CCK-8 assay (Figure 11C). In addition, wound healing assays and transwell assays were performed to examine cell migration and invasion. After silencing of circEYA1, cells showed decreased ability in cell migration and invasion (Figures 11D,E). Cell cycle distribution analysis showed that the proportion of cells in G1-phase was decreased significantly and that the G2-phase and S-phase proportion were increased following silencing of circEYA1 (Figure 11F). It is well known that circRNAs could serve as miRNA sponges, indirectly regulating the expression of downstream mRNAs. Correlation analysis revealed that the expression of TP53 mRNA showed a positive correlation with circEYA1 (Figure 11G). Forecasting the circRNA/miRNA/mRNA axis of circEYA1, we found that miR-619-5p could bind to circEYA1 and TP53 was one of the potential targets. The possible binding sites are shown in Figure 11H. In addition, circEYA1 silencing significantly increased the expression of miR-619-5p, while decreased TP53 expression (Figure 11I). These results suggest the potential role of the constructed ceRNA network in WT carcinogenesis.

## Discussion

Comprehensive treatment of pediatric WT has resulted in excellent outcomes, yet the underlying molecular mechanisms driving pathogenesis and tumor progression remain poorly defined, particularly in the context of circRNAs. Therefore, it is of great importance to explore the molecular mechanisms underlying WT development and identify effective biomarkers that have significant implications for better treatment and better prognosis. There has been substantial interest in the ceRNA hypothesis in recent years. Based on the ceRNA hypothesis, circRNAs could act as ceRNAs to interact with mRNAs by competing with the corresponding miRNAs (Hansen et al., 2013; Memczak et al., 2013). Increasing evidence suggests that circRNA and circRNA-mediated ceRNA networks play a crucial role in the pathogenesis and progression of various cancers (Zhong et al., 2018). For instance, hsa_circ_0014130 works as a sponge of miR-132-3p to advance the oncogenesis and metastasis of bladder cancer by regulating KCNJ12 expression (Li et al., 2021), and circAGFG1 acts as a sponge of miR-195-5p to promote triple-negative breast cancer progression by regulating CCNE1 expression (Yang et al., 2019). Here, to explore the underlying mechanisms of the circRNA-mediated ceRNA network involved in the occurrence and progression of WT, we constructed a circRNA-miRNA-mRNA ceRNA regulatory network based on sequencing data of clinical samples.

To date, no comprehensive expression profile of DE-circRs has been reported in WT. In this study, we systematically analyzed the circRNA expression profile in WT by next-generation sequencing technology. More than half of these circRNAs (54.61%) were newly identified when compared with the known database circBase. The circRNA species and their chromosomal distribution were similar to those in other tumors (Xu et al., 2019). A total of 314 DE-circRs (115 upregulated and 199 downregulated) were identified according to the screening criteria described in the method. To validate the DE-circR profiling, four DE-circRs were randomly selected and validated with qRT–PCR, and the expression trend was consistent with that in the sequencing results. These results were further confirmed by Sanger sequencing of the PCR products. In addition, we analyzed the DE-mRs between WT and normal tissues. Interestingly, most DE-circRs were downregulated, whereas most DE-mRs were upregulated in WT. These results indicated that a potential complex regulatory ceRNA network exists.

GO and KEGG enrichment analyses of DE-circR parental gene showed the enrichment in many cancer-related pathways involved in “cell proliferation”, “cell cycle”, and several other malignant biological processes. GO-CC indicated that they were localized mostly to the cytoplasm. Generally, most circRNAs derived from exons were found to be predominantly localized in the cytoplasm and to play important roles in transcriptional regulation. Notably, these circRNAs were also abundant in the “extracellular exosome” component. Recently, circRNAs have been identified for their enrichment and stability in exosomes (Wang et al., 2019). Moreover, a growing body of research has illuminated that several circRNAs derived from exosomes act as prognostic biomarkers and regulate tumor cell proliferation, invasion, metastasis, and chemoresistance (Wang X. et al., 2020; Ding et al., 2020; Huang et al., 2020; Shang et al., 2020; Xie et al., 2020). In this way, exosome-derived circRNAs could participate in the regulation of the tumor microenvironment in WT. GO and KEGG pathway analyses were also used to generate functional annotations for DE-MRs in WT. The results showed that these DE-mRs were involved in cell division, the cell cycle, proliferation, metabolism, and the classical p53 pathway, and these signaling pathways have been proven to regulate function and fate. These results support the pivotal role of these DE-circRs and DE-mRs in cancer-associated pathways and further indicate that the circRNA-related ceRNA network could be involved in the initiation and progression of WT.

Studies have discovered that circRNAs serve as miRNA sponges to indirectly regulate gene expression. To reveal the detailed functions and mechanisms of these circRNAs in WT, we integrated the circRNA-miRNA interactions and miRNA-mRNA interactions and constructed a circRNA-miRNA-mRNA ceRNA regulatory network. To explore the potential biological function of mRNAs in the ceRNA network, GO and KEGG pathway enrichment analyses of mRNAs were also performed. The results indicated that these mRNAs were mainly enriched in cell division, molecule binding, the p53 signaling pathway, the cell cycle, and the Hippo signaling pathway. GSEA was used to assess the group behavior of a set of genes rather than assessing individual genes. The evaluation regarding outcomes identified mRNAs were mainly related to cell cycle modulation, agreeing with the above results. Notably, these mRNA targets were significantly enriched in the NK cell pathway, whereas NK cell activity was correlated with WT patient survival. Consistent with the above findings, these results indicate that these mRNAs within the ceRNA network may regulate tumor growth and progression via the cell cycle and immune pathways.

In the search key regulatory networks, PPI network analysis using the STRING database revealed the top ten hub genes. With the exception of KANK3, which was downregulated, the others were upregulated. Furthermore, by performing survival analyses, we demonstrated that WT patients with high levels of TP53, KANK3, LLGL1, and TOP3A expression have poor prognosis using the univariate Cox analysis model. This further supports a potential role for these hub genes in WT biological implications. By searching PubMed and Web of Science, we found possible mechanisms of these hub genes in tumorigenesis. The TP53 gene encodes a tumor suppressor protein containing transcriptional activation, DNA binding, and oligomerization domains. The encoded protein responds to diverse cellular stresses to regulate the expression of target genes, thereby inducing cell cycle arrest, apoptosis, senescence, DNA repair, or changes in metabolism (Yin et al., 2002; Marcel et al., 2010). Aberrant expression of TP53 has been detected in multiple cancers and is useful as a diagnostic and prognostic marker in cancer progression (Sun et al., 2020). Mutations in this gene are also potential prognostic and predictive markers, as well as targets for pharmacological intervention (Olivier et al., 2010; Bykov et al., 2018). Our study reached similar conclusions that TP53 mutations are predictive of patient survival and demonstrate significant associations with adverse pathological type. These findings reveal TP53 as a potential therapeutic target in WT patients. TP53 has also been reported as a downstream target of circRNAs. For example, circCNTNAP3 inhibits proliferation and promotes apoptosis in p53 wild-type esophageal squamous cell carcinoma (ESCC) cells; mechanistically, circCNTNAP3 promotes the expression of p53 by sponging miR-513a-5p (Wang H. et al., 2020).

Human KANK family proteins (KANK1, 2, 3, and 4) have been described as essential for crosstalk between actin and microtubules (MTs) (Chen et al., 2018). As such, these proteins are mediators of cell migration, proliferation, and apoptosis (Tadijan et al., 2021). KANK1 was among the first found to be downregulated in a variety of tumors and has been identified as a tumor suppressor (Sarkar et al., 2002; Chen et al., 2017; Fan et al., 2020). Low KANK1 expression in tumor tissues was associated with a poor prognosis in oral squamous cell carcinoma (OSCC), astrocytoma, and lung adenocarcinoma patients (Kim et al., 2018; Fan et al., 2020), while KANK1 overexpression indicated a good prognosis in patients with invasive breast cancer (Kariri et al., 2020). KANK1 has also been reported as a target for circRNA-sponge mechanisms; for example, circDDX17 reduces 5-fluorouracil resistance and hinders tumorigenesis in colorectal cancer by regulating the miR-31-5p/KANK1 axis (Ren et al., 2020). Relatively, KANK3 is less studied. Kim et al. found that KANK3 downregulation enhanced the migration and invasion ability of hepatocellular carcinoma cells. Bioinformatics analysis revealed their association with a poor prognosis in multiple cancer types (Kim et al., 2018). Interestingly, KANK3 has been shown to be a hypoxia-induced proapoptotic target for p53 (Leszczynska et al., 2015). Therefore, the KANK3 protein might be a candidate for improving cancer therapy.

LLGL1 is also known as Lgl1, HUGL1, and HUGL-1. LLGL1 has been implicated to play oncogenic roles in various types of malignancies, such as pancreatic ductal carcinoma (Zhu et al., 2020), non-small-cell lung cancer (Yang et al., 2016), gliomas (Liu et al., 2015), and esophageal cancer (Song et al., 2013), by regulating cell proliferation, invasion, apoptosis, and drug resistance. Moreover, LLGL1 has already been proposed as a prognostic marker of various cancers (Tsuruga et al., 2007; Lu et al., 2009; Biesterfeld et al., 2012). TOP3A encodes a DNA topoisomerase, an enzyme that controls and alters the topologic states of DNA during transcription. Several studies have reported the role of TOP3A in cancer. It interacts with p53 and contributes to p53-mediated tumor suppression (Hsieh et al., 2014). Another study found a correlation between its expression and cancer stemness or immune phenotype in oral tongue squamous cell cancer (OTSCC), but this gene failed to show prognostic significance in OTSCC (Oliveira-Costa et al., 2012). Collectively, we identified several promising targets that may play a critical role in WT development and deserve further investigation.

Although most children with WT have a relatively good prognosis, a subset of patients subsequently experience disease relapse. Previous lessons indicate that these high-risk patients should be treated aggressively (Spreafico et al., 2009; Malogolowkin et al., 2013). The current COG approach to these patients is treatment on clinical staging and anaplasia pathological types (Ko and Ritchey 2009). Existing strategies, however, may not be accurate enough, given that many cases of relapse have been found in patients without any adverse risk (Brok et al., 2018). Therefore, delineating accurate predictors of progression is crucial to identifying the subset of patients who need more aggressive treatment. In this study, we identified four genes associated with DFS, based on the present circRNA-related network. Next, a prognostic gene signature consisting of three genes was constructed; it successfully classified patients into high-risk and low-risk groups and could accurately predict patient prognosis. The above research results illustrated that the gene signature might serve as a prognostic biomarker in clinical application and therefore might be useful to help improve individualized treatment planning. Finally, we established a key circRNA-mediated regulatory subnetwork based on the importance of the gene signature. qRT–PCR was used to verify the differential expression of the top-ranked three circRNAs along with the three-gene signature. Moreover, functional analysis revealed that silencing of circEYA1 suppressed proliferation, invasion, and migration in WT cells, suggesting that circEYA1 exerts an oncogenic effect in WT. In mechanism, our results indicate that circEYA1 might function by molecular sponge, a ceRNA mechanism with TP53 and miR-619-5p. These findings are powerful proofs of the indispensable roles of circRNAs in WT. However, the specific mechanisms by which these genes regulate WT cell biological functions are still insufficient and further experimental validation is needed.

Accumulating evidence reveals that the immune microenvironment plays an essential role in tumor development and therapy (Quail and Joyce 2013; Hinshaw and Shevde 2019). The tumor microenvironment is a complex mix of structural components and inflammatory and immune cells. Immune cells are the fundamental component of the tumor microenvironment and might be one of the important aspects of the tumor immune escape mechanism. Of these immune cells, NK cells and CD8^+^ T cells play a crucial role in eradicating cancer cells, and inhibition of their functions is a key mechanism of tumor immune escape (Farhood et al., 2019; Khan et al., 2020). The functional activity of NK and CD8^+^ T cells is, therefore, an important research area in antitumor immunotherapy. As described in the Results section, NK cells and CD8^+^ T cells, as key immune factors, play critical anticancer roles and affect the long-term survival rates of WT patients, suggesting their potential application in predicting the response to immunotherapy in WT. Similarly, circRNAs can also act as immune regulators in the development and progression of cancer (Chen 2020). For example, in hepatocellular carcinoma, circUHRF1 inhibits NK cell function by upregulating the expression of TIM-3 via degradation of miR-449c-5p (Zhang et al., 2020). In non-small-cell lung cancer, circCPA4 promoted cell death by downregulating PD-L1 through serving as an RNA sponge for let-7 miRNA and reactivated CD8^+^ T cells in the coculture system positively via regulated exosomal PD-L1 (Hong et al., 2020). These results suggest a potential mechanism for the association between circRNAs and immune regulation, and circRNAs may be pivotal regulators in the process. From this, we explored the role of the three hub genes within the circRNA-related network in the tumor immune microenvironment of WT. Differential expression analysis revealed that immune checkpoint-related genes were differentially expressed in the high and low expression groups of the three hub genes. Interestingly, high expression of TP53 limited the efficacy of ICB, leading to immune escape. Overall, the TP53 gene plays a substantial role in WT and is expected to be an attractive biomarker and therapeutic target. On the basis of the potential importance of TP53 in WT pathology, we confirmed its expression in WT not only by RT–PCR but also by WB analysis.

To our knowledge, the ceRNA regulatory network, prognostic gene signature, and immune markers derived from the present study have not been previously reported. Findings from our study support a potential new target for WT therapy and prediction. In addition, we validated the expression of key genes using clinical samples and WT cell lines. Besides these, we selected a key circRNA, circEYA1, for function validation. Nevertheless, there are several limitations to this study. First, there were no samples with miRNA sequencing data, so the miRNAs within the ceRNA network require appropriate experimental validation. Second, these newly identified ceRNA relationships were based on a bioinformatics approach, and further experimental validation of this hypothesis is required. Finally, further *in vitro* and *in vivo* experiments are necessary to verify the biological function including how it is involved in immune regulation of the ceRNA network. We will confirm this hypothesis in our future experiments.

In summary, in this study, we described for the first time the circRNA expression profiles in WT and established a circRNA-related ceRNA regulatory network involved in tumor progression and the regulation of immune responses. These findings provide new insights into the mechanisms of WT and propose potential therapeutic targets deserving further exploration and verification. Additionally, a prognostic model consisting of three prognosis-associated hub genes was constructed based on the circRNA-related network, and this model could be used to guide the individualized treatment of WT patients.

## Data Availability

The datasets presented in this study can be found in online repositories. The name of the repository and accession numbers can be found below: NCBI; GSE197046 and GSE197047.
