# Supplementary material for: The Regulatory Network and Role of the circRNA-miRNA-mRNA ceRNA Network in the Progression and the Immune Response of Wilms Tumor Based on RNA-Seq
Source: Front Genet. 2022 Apr 26;13:849941. doi: 10.3389/fgene.2022.849941 (PMC9086559; doi:10.3389/fgene.2022.849941)
Supplement: Supplementary file 3 [file Table1.DOCX]

**Supplementary Table 1. Primers used for quantitative real time PCR**

| RNA | Forward primer | Reverse primer |
| --- | --- | --- |
| hsa_circ_0001900 | CGTTCAGTGCCTCGAAAGAAC | CTGGTCCCCTTTCAGGATGAG |
| hsa_circ_0009035 | TTGCCTCTGGAGTATTGGAGT | AAGCTAATGTGTCCCTGTTGG |
| circNPNT | AAGTTGGAGAGAGAAGGGAAC | TGGTCAGGTTTCTACTGTTATTTTG |
| hsa_circ_0072391 | GCTCGGATGTTGCTGAATGAC | AGCAGCGGTCTAATGCACTG |
| circEYA1 | CCTTGGACTGCGAATGGAAGA | TCTGAACCTCGACGCAATCG |
| circZNF208 | CAGAGCAGGGCATAGAAGATT | TTCTATGGCCACATCCCTAAA |
| circPKHD1 | TTGGATCTTACTCGTGTGCC | AAAGGAATCCACTTACCCTGG |
| TP53 | CGCTTCGAGATGTTCCGAGA | CTTCAGGTGGCTGGAGTGAG |
| LLGL1 | GGAACCTGGCAGAAGACGAG | AGCAGGCGTGGTTCTATGTT |
| KANK3 | CTCCGCTAGCGACATTCCAG | GGTCGGGCAGGTTCTGATT |
| GAPDH | CCTTCCTGGGCATGGAGTC | TGATCTTCATTGTGCTGGGTG |
