# Supplementary material for: The Regulatory Network and Role of the circRNA-miRNA-mRNA ceRNA Network in the Progression and the Immune Response of Wilms Tumor Based on RNA-Seq
Source: Front Genet. 2022 Apr 26;13:849941. doi: 10.3389/fgene.2022.849941 (PMC9086559; doi:10.3389/fgene.2022.849941)
Supplement: Supplementary file 4 [file Table4.DOCX]

**Supplementary Table 2. Basic characteristics of selected DE-circRNAs**

| circRNA | Position | Strand | Genomic length | Spliced seq length | Host gene ID | Gene symbol | Regulation |
| --- | --- | --- | --- | --- | --- | --- | --- |
| hsa_circ_0009035 | chr12:49991998-49992655 | − | 658 | 375 | ENSG00000161800 | RACGAP1 | Up |
| circNPNT^†^ | chr4:105911149-105911732 | + | 584 | 584 | ENSG00000168743 | NPNT | Down |
| hsa_circ_0001900 | chr9:135881633-135883078 | − | 1446 | 425 | ENSG00000130559 | CAMSAP1 | Up |
| hsa_circ_0072391 | chr5:43295752-43297166 | − | 1415 | 331 | ENSG00000112972 | HMGCS1 | Down |
| circZNF208^†^ | chr19:21974729-21988909 | − | 14181 | 302 | ENSG00000160321 | ZNF208 | Down |
| circEYA1^†^ | chr8:71244603-71271897 | − | 27295 | 314 | ENSG00000104313 | EYA1 | Up |
| circPKHD1^†^ | chr6:52010309-52035721 | − | 25413 | 638 | ENSG00000170927 | PKHD1 | Down |

†circRNAs, without circbase (https://www.circbase.) annotations, were provided as annotated in the circAtlas 2.0 database (http://circatlas.biols.ac.cn/) with a circAtlas ID (circNPNT = hsa-NPNT_0006, circZNF208 = hsa-ZNF208_0001, circEYA1 = hsa-EYA1_0007, circPKHD1 = hsa-PKHD1_0007) for detail information.
